# Photon-Counting CT Enables Higher Image Quality at a Lower Radiation Dose Compared with a 256-Slice Energy-Integrating Detector CT in Pediatric Patients with Congenital Heart Disease

**DOI:** 10.3390/diagnostics16050735

**Published:** 2026-03-01

**Authors:** André Lollert, Fabio Souschek, Tariq Abu-Tair, Frank Dette, Georg Daniel Duerr, Christoph Kampmann, Tobias Bäuerle, Gundula Staatz

**Affiliations:** 1 Department of Diagnostic and Interventional Radiology, Section of Pediatric Radiology, Medical Center of the Johannes Gutenberg-University, 55131 Mainz, Germany; 2 Center for Diseases in Childhood and Adolescence, Division of Pediatric Cardiology and Congenital Heart Diseases, Medical Center of the Johannes Gutenberg-University, 55131 Mainz, Germany; 3 Department of Anesthesiology, Medical Center of the Johannes Gutenberg-University, 55131 Mainz, Germany; 4 Department of Cardiovascular Surgery, Section of Congenital Heart Surgery, Medical Center of the Johannes Gutenberg-University, 55131 Mainz, Germany; 5 Department of Diagnostic and Interventional Radiology, Medical Center of the Johannes Gutenberg-University, 55131 Mainz, Germany

**Keywords:** congenital heart disease, photon-counting CT, radiation dose, pediatric cardiology, pediatric radiology

## Abstract

**Background/Objectives**: There is an increasing need for cross-sectional imaging in pediatric patients with congenital heart disease. This patient group is highly sensitive to ionizing radiation. The purpose of this article was to evaluate differences in radiation dose and image quality between a first-generation photon-counting (PC)-CT system and a 256-slice single-source energy-integrating detector (EID)-CT in these patients. **Methods**: We retrospectively assessed effective dose, CT dose index-volume (CTDI_vol_), dose length product (DLP), and image quality of all prospectively electrocardiography-gated CTs of the thorax in all patients < 18 years of age examined between February 2021 and August 2024 (n = 43). Two independent observers subjectively scored image quality, vascular contrast, and noise on a 5-point Likert scale. In addition, we assessed the signal-to-noise-ratio (SNR) and contrast-to-noise-ratio (CNR) quantitatively. **Results**: All dose parameters were significantly lower in the PC-CT (n = 27) versus the EID-CT (n = 16) group (mean effective dose: 0.8 ± 0.64 versus 2.2 ± 0.88 mSv, *p* < 0.001; mean CTDI_vol_: 1.22 ± 0.96 versus 4.8 ± 1.08 mGy, *p* < 0.001; mean DLP: 30.7 ± 31.9 versus 73.7 ± 50.7 mGy*cm, *p* < 0.001). Overall subjective image quality and contrast were rated higher in the PC-CT group (*p* = 0.046 and < 0.001, respectively). Quantitative CNR was significantly higher in the PC-CT group (mean 39.1 ± 12.9 versus 26.2 ± 10.8, *p* = 0.002). **Conclusions**: PC-CT enables high-quality examinations for the evaluation of congenital heart disease with a highly significant dose reduction compared with a 256-slice single-source EID-CT.

## 1. Introduction

Congenital heart disease (CHD) is one of the most common causes of morbidity and mortality in neonates and infants [1,2]. The primary imaging modality to screen for CHD and to evaluate these children pre- and postnatally is ultrasound [3,4]. However, in complex cases, especially those requiring surgery early in life, ultrasound is unable to depict the entire cardiovascular anatomy in detail [5]. Magnetic resonance imaging (MRI) has the advantage of being radiation-free and enables the acquisition of dynamic and functional data. Still, the drawbacks of this modality compared with computed tomography (CT) include longer examination times, often necessitating anesthesia and intubation, and lower spatial resolution. Especially in neonates, MRI is unable to depict small vascular structures in detail [6], and the risk of anesthesia prohibits lengthy MRI examinations in these fragile patients. Therefore, CT has emerged as an important modality for the preoperative evaluation of patients with CHD, sometimes in addition to MRI and ultrasound [7]. Furthermore, many patients with CHD require follow-up imaging for future operative or endovascular interventions, also later in life [8], potentially leading to a high cumulative X-ray exposure [9]. Thus, the minimization of radiation dose is crucial, especially in pediatric patient collectives. In the past, a first step towards dose reduction for cardiac imaging was the introduction of prospectively electrocardiogram (ECG)-gated sequential scanning (the “step-and-shoot” technique), for example, with last-generation 256- or 320-slice single-source CT scanners [10]. More recent dual-source scanners allowed for further dose reductions using high-pitch scan modes [11].

While the aforementioned conventional energy-integrating detector (EID)-CTs convert X-ray photons into visible light indirectly, photon-counting (PC)-CT measures the specific energy of every photon directly. This leads to a lower signal loss, an improved iodine contrast-to-noise ratio (CNR), and enables the gain of spectral information [12,13]. Consequently, the technology allows for the generation of virtual monoenergetic images (VMIs) at different levels. Of these, especially low-keV reconstructions are suitable to enhance vascular contrast [14,15]. This applies in particular to neonates and infants, in whom high flow rates for the injection of contrast media cannot be achieved due to small catheter sizes [15].

In pediatric patients with CHD, PC-CT has shown a higher image quality at a similar dose compared with a third-generation dual-source EID-CT [16]. However, in the aforementioned study, most of the patients were below 1 year of age, with no patient being older than 3 years. In another study, a high diagnostic image quality was achieved with low radiation doses at both 70 and 90 kV PC-CT [17]. Comparative studies with sequential EID-CT scan modes (“step-and-shoot”) have not been published yet. Therefore, the purpose of this study was to compare dose and image quality of PC-CT versus a 256-slice single-source EID-CT in a broad collective of pediatric patients with CHD.

## 2. Patients and Methods

### 2.1. Patients

Approval by the local Independent Ethics Committee was not necessary according to local regulations due to the retrospective study design. At our institution, a new section of congenital heart surgery was established in 2021, which led to an increase in pediatric cardiovascular CT examinations. Therefore, the study population consisted of all pediatric (i.e., <18 years of age) patients with CHD who underwent an ECG-gated CT angiography covering the whole thorax from February 2021 until August 2024. At the beginning of this time frame, the examinations were performed on the EID-CT, while after its installation, and after the installation of the corresponding pediatric anesthesia unit in 2022, the majority of examinations were performed on the PC-CT. We excluded patients in whom only the heart and coronary arteries were scanned, because of the shorter scan length (e.g., fewer necessary steps in the sequential scan mode).

### 2.2. CT Scans and Dose Assessment

The legal guardians of all patients provided written informed consent for the clinically indicated CT examinations.

We applied sedation in small children depending on patient compliance. Examinations in neonates (i.e., children < 3 months of age) were conducted using the “feed-and-wrap” technique [18] using an immobilizing vacuum mattress, if possible, due to the general patient condition. The decision whether to use sedation or not was made by the radiologist, anesthesiologist, and pediatric cardiologist in consensus. In cases in which a successful examination was clinically necessary without delay, we swaddled the child without prior feeding to be able to switch to sedation immediately if patient movement was too severe. We performed the EID-CT examinations on a 256-slice single-source scanner (Brilliance iCT 256, Philips Healthcare, Eindhoven, The Netherlands) and the PC-CT examinations on the first commercially available system (Naeotom Alpha, Siemens Healthineers, Erlangen, Germany). EID-CT scans were acquired with a tube potential of 80 kVp, PC-CT scans with a tube potential of 70 kVp in young children (<10 years). In children above 10 years of age, a 120 or 140 kVp multi-energy spectral acquisition mode (QuantumPlus, Siemens Healthineers) was used. In all examinations, imaging was performed with prospective ECG-gating. We triggered at 40% of the R-R interval because of the higher heart rates in pediatric populations. ECG-electrodes were placed outside the scan field (e.g., on the upper arms instead of the thorax) to avoid artefacts, whenever possible. On the EID-CT, sequential scanning (“step-and-shoot” mode) was performed, whereas on the PC-CT we used a fast rotation, high-pitch mode (Turbo-Flash-Mode, Siemens Healthineers). Table 1 summarizes further technical details about the acquisition and reconstruction parameters.

Contrast media protocols were identical for both CT scanners: In neonates and infants, we applied 2 mL/kg body weight of iodinated contrast agent (Accupaque 300, GE Healthcare, Chicago, IL, USA), followed by 10 mL of saline by manual injection. The examination was started immediately after the injecting physician had left the scanner room. In older children, we used an automated pump for contrast media injection (1.5 mL/kg body weight, Ultravist 370, Bayer Vital, Leverkusen, Germany) at a flow rate of 3–5 mL/s, depending on the size of the i.v. tube. Contrast injection was followed by 20–50 mL of saline at a flow rate of 2–4 mL/s. Either a test bolus or bolus tracking in the ascending aorta was used to determine the start of the scan in these latter cases.

For our further analyses, we collected the CT dose index (CTDI_vol_) and dose length product (DLP) from the dose data sheets. A 32-cm PMMA body phantom was used to calibrate. In addition, we documented the effective dose, as assessed by our dose management system (DOSE, Qaelum Inc., New York, NY, USA). This software uses the age-specific conversion factors from IRCP 103 [19].

### 2.3. Qualitative Image Analysis

Two observers (one pediatric radiologist with more than 10 years of experience in pediatric CT and cardiovascular imaging, AL, and one 4^th^-year radiology resident, FS) independently assessed image quality. A 5-point Likert scale was applied to evaluate overall image impression (5 = excellent, 4 = good, 3 = moderate, 2 = bad, 1 = non-diagnostic), vascular contrast (5 = excellent, 4 = above average, 3 = acceptable, 2 = suboptimal, 1 = very poor), and image noise (5 = no/very little, 4 = little, 3 = moderate, 2 = strong, 1 = very strong). The observers were principally blinded to which scanner was used for these assessments. However, the image impression (e. g., sharper vessel edges on the PC-CT) enables radiologists working with both machines to identify the scanner, which made blinding not completely possible.

### 2.4. Quantitative Image Analysis

We also assessed signal-to-noise ratio (SNR) and CNR, as described before [16]. We analyzed reconstructions with a slice thickness of 0.8 mm for both scanners; in the case of PC-CT scans, 40 keV VMIs were used for the quantitative measurements. SNR was calculated as the mean attenuation (in Hounsfield units) of 3 regions of interest (ROI) in the ascending aorta, the aortic arch, and the descending aorta, divided by the mean standard deviation. We defined contrast as mean vascular attenuation (as described before) minus mean attenuation of subcutaneous fat. CNR was calculated by dividing the contrast by noise (standard deviation in a ROI in the subcutaneous fat). All ROIs were placed, including the maximum vascular diameter, with a minimum size of 10 mm^2^. ROIs in the subcutaneous fat were placed, avoiding areas with artefacts, for example, caused by ECG-electrodes or external wires.

### 2.5. Statistics

Statistical evaluations were performed with SPSS Statistics (Statistical Package for the Social Sciences, version 29.0; IBM, Armonk, NY, USA). We used the Mann-Whitney U test to assess differences in age and body mass index as well as dose and image quality parameters between the EID-CT and PC-CT groups. The Chi-squared test was applied to test for gender differences. Due to the age heterogeneity of our collective, we performed subgroup analyses based on patient age. Subgroup 1 consisted of patients with an age of 0 to 24 months, subgroup 2 included patients between 2 and 9 years of age, and subgroup 3 included patients ≥ 10 years of age. Inter-observer agreement for the subjective and quantitative image quality analyses was evaluated using the intra-class correlation coefficient (ICC, two-way mixed model, absolute agreement) [20]. A *p*-value of less than 0.05 was considered significant in all analyses, which were exploratory; no adjustments for multiple testing were made.

## 3. Results

**Figure 1:**
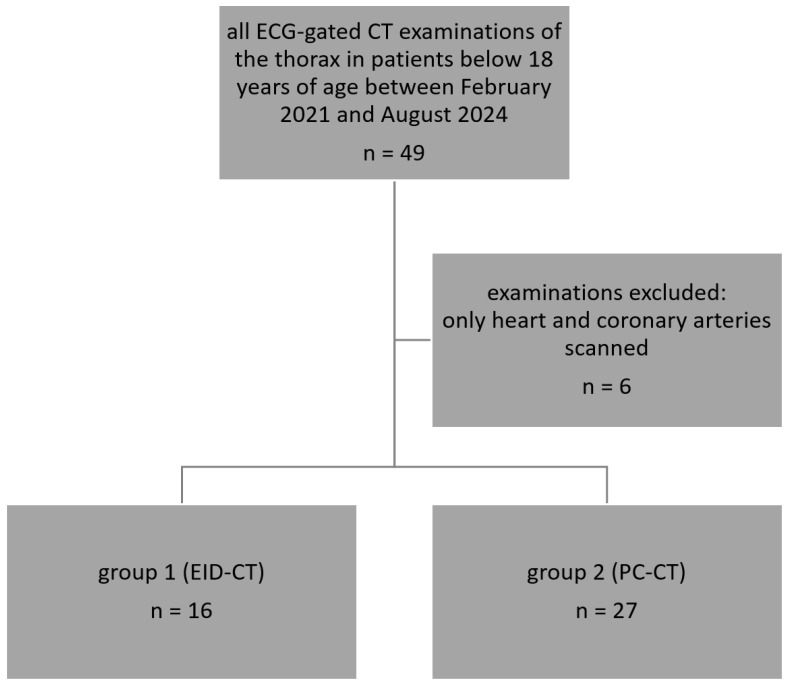
Flow chart of the included and excluded examinations.

Six out of the 49 cardiovascular CT examinations in the study period were excluded, as only the heart and coronary arteries were scanned. Thus, the final study cohort consisted of 43 scans (Figure 1) in 40 patients (20 male, 20 female).

3/40 patients had two CT scans during the study period. Diagnoses included aortic coarctation/hypoplastic aortic arch (n = 12), atrial and/or ventricular septal defect (n = 4), pulmonary atresia, hypoplastic left heart, congenital pulmonary airway malformation/pulmonary sequestration (n = 3, respectively), common arterial trunk, aortopulmonary window, sinus venosus defect, (n = 2, respectively), double-outlet right ventricle, tetralogy of Fallot, ectopia cordis, coronary fistula, d-transposition of the great arteries, aneurysm of a patent ductus arteriosus, aortic valve stenosis (status after Ross-operation), Shone complex, and pulmonary sling (n = 1, respectively). Patient characteristics and differences between the EID-CT and PC-CT groups for the whole collective, as well as for subgroups 1–3, are shown in Table 2.

Age, body weight, body surface area, and body mass index did not significantly differ between the PC-CT and EID-CT groups in the whole collective as well as in all subgroups. In subgroup 1, there was a significant difference in the gender distribution (no females were scanned using the EID-CT).

### 3.1. Dose Comparison

**Figure 2:**
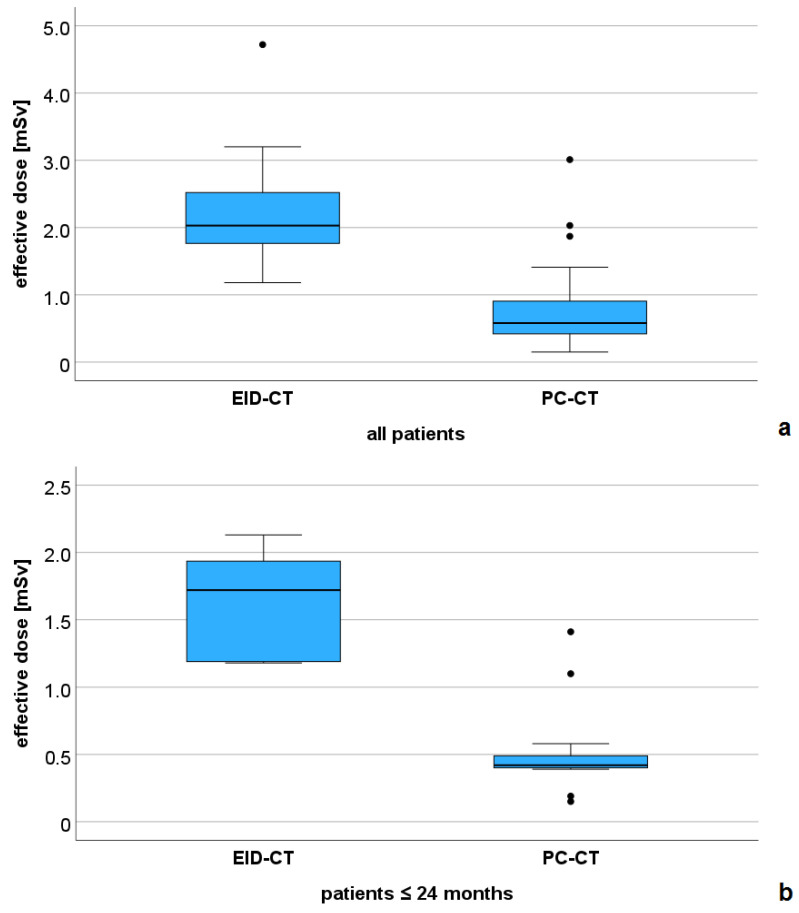
Comparison of the effective dose between the EID-CT and PC-CT groups. (**a**) all patients (n = 43), and (**b**) subgroup of patients with a maximum age of 24 months (n = 20).

All dose parameters were significantly lower in the PC-CT versus the EID-CT group. For CTDI_vol_ mean ± SD was 1.22 ± 0.96 versus 4.8 ± 1.08 mGy (*p* < 0.001), for DLP mean ± SD was 30.7 ± 31.9 versus 73.7 ± 50.7 mGy*cm, and for the effective dose mean ± SD was 0.8 ± 0.64 versus 2.2 ± 0.88 mSv (*p* < 0.001, Figure 2a). In the PC-CT group, 20/27 examinations (74%) had an effective dose below 1 mSv. All subgroup analyses also demonstrated a lower effective dose for the PC-CT examinations (subgroup 1: mean ± SD: 0.53 ± 0.35 versus 1.61 ± 0.42 mSv, *p* < 0.001, Figure 2b, subgroup 2: mean ± SD: 0.65 ± 0.18 versus 2.06 ± 0.23 mSv, *p* = 0.003, and subgroup 3: mean ± SD: 1.8 ± 0.85 versus 3.14 ± 0.94 mSv, *p* = 0.056).

The DLP was also lower in all PC-CT subgroups (subgroup 1: mean ± SD: 16.08 ± 12.6 versus 46.5 ± 13.5 mGy*cm, *p* = 0.001, subgroup 2: mean ± SD: 23.1 ± 6.3 versus 71 ± 17.1 mGy*cm, *p* = 0.003, and subgroup 3: mean ± SD: 82.3 ± 43.6 versus 137 ± 51.4 mGy*cm, *p* = 0.151)

**Figure 3:**
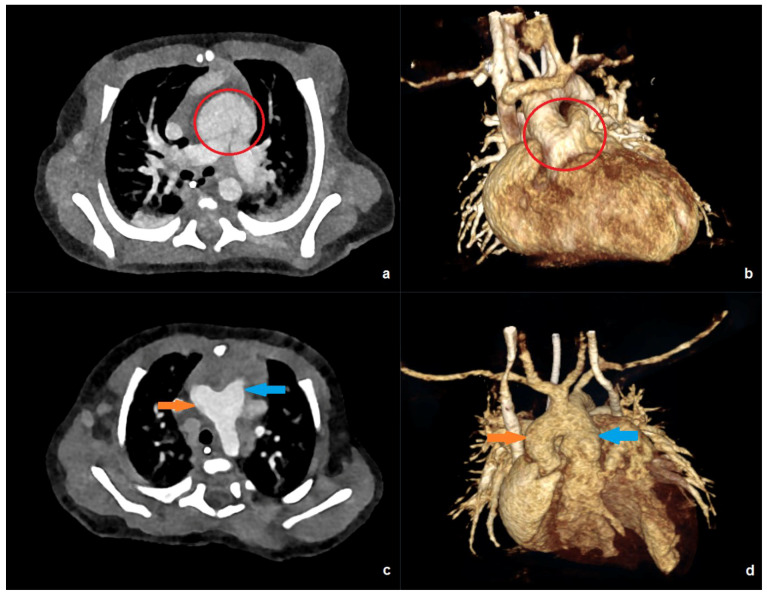
PC-CT of a 10-day-old patient with an effective dose of 0.19 mSv: (**a**) transverse maximum intensity projection, and (**b**) 3D anterior reconstruction demonstrating a common arterial trunk (red circle). PC-CT of a 59-day-old patient with an effective dose of 0.41 mSv: (**c**) transverse image, and (**d**) 3D anterior reconstruction showing a large aorto-pulmonary window with a connection between the pulmonary trunk (blue arrow) and the ascending aorta/aortic arch (orange arrow).

Figure 3 shows examples of low-dose PC-CT examinations in two of the youngest patients in our collective.

### 3.2. Image Quality Comparison

Inter-observer agreement was moderate for all qualitatively assessed categories (overall image impression: ICC = 0.576, 95%-confidence interval [CI]: 0.224–0.77, *p* = 0.001; vascular contrast: ICC = 0.638, 95%-CI: 0.33–0.804, *p* < 0.001; and noise: ICC = 0.61, 95%-CI: 0.291–0.787, *p* < 0.001).

The overall subjective image quality, averaged between the two observers, was significantly higher in the PC-CT versus the EID-CT group (mean ± SD: 4.4 ± 0.39 versus 4.1 ± 0.43, *p* = 0.046), as well as the vascular contrast rating (mean ± SD: 4.7 ± 0.47 versus 4.2 ± 0.44, *p* < 0.001). The noise rating did not significantly differ between the two groups (mean ± SD: 3.8 ± 0.39 versus 3.9 ± 0.65, *p* = 0.36).

**Figure 4:**
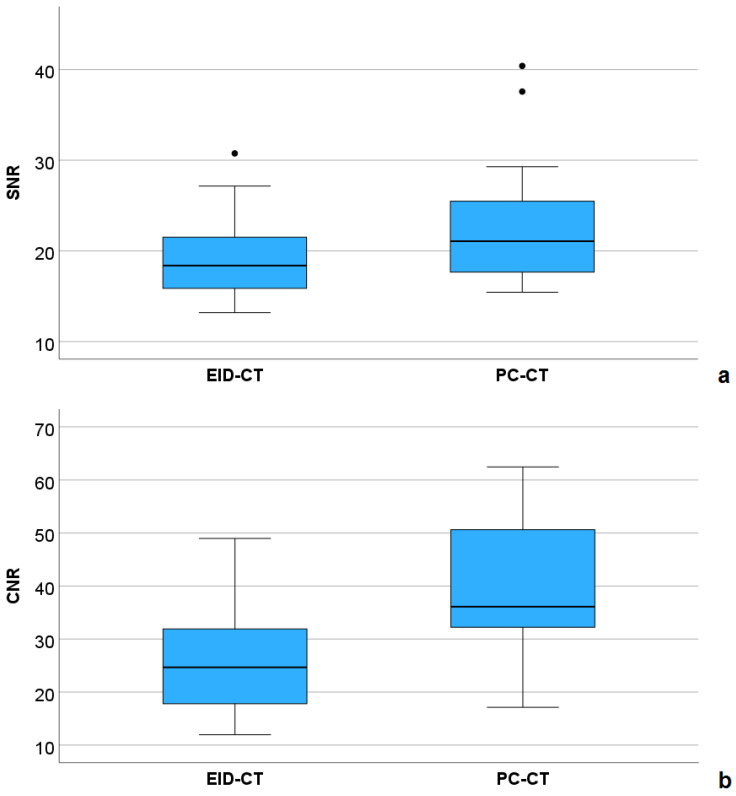
Comparison of signal-to-noise ratio (SNR, (**a**)) and contrast-to-noise ratio (CNR, (**b**)) between the EID-CT and PC-CT group.

Inter-observer agreement was excellent for SNR (ICC = 0.986, 95%-CI: 0.975–0.993, *p* < 0.001) and CNR (ICC = 0.955, 95%-CI: 0.917–0.976, *p* < 0.001). Both quantitative parameters of image quality were higher in the PC-CT compared with the EID-CT group (Figure 4).

**Figure 5:**
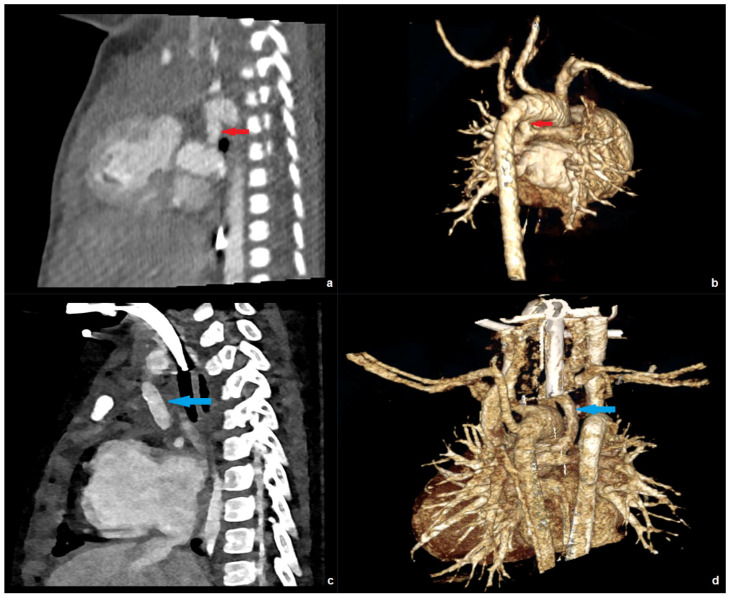
EID-CT images of a patient with hypoplastic left ventricle and pulmonary atresia at the age of 10 days: sagittal (**a**) and 3D right posterior oblique (**b**) reconstruction depicting the patent ductus arteriosus (red arrow). PC-CT images of the same patient at the age of 17 month sharply depicting a Blalock-Taussig shunt (blue arrow) in the sagittal (**c**) and 3D right posterior oblique (**d**) view.

For CNR, this was statistically significant (mean ± SD: 39.1 ± 12.9 versus 26.2 ± 10.8, *p* = 0.002), for SNR, a non-significant equivalent tendency was demonstrated (mean ± SD: 22.5 ± 6.3 versus 19.3 ± 5.2, *p* = 0.097). Figure 5 shows an example of a female patient with hypoplastic left ventricle and pulmonary atresia, who underwent EID-CT at the age of 10 days, and PC-CT at the age of 17 months, both under sedation. In the latter examination, a higher CNR (34.6 versus 16.4), leading to a better vascular delineation as well as sharper 3D reconstructions, could be achieved with a lower effective dose (0.42 versus 1.18 mSv).

## 4. Discussion

In this study, we demonstrated a significant intra-institutional dose reduction when using PC-CT compared with 256-slice EID-CT for the pre-operative or pre-interventional evaluation of a broad collective of patients with CHD. Especially in the most radiation-sensitive patients (subgroup 1, 0–24 months of age), examinations could be performed with sub-mSv doses in nearly all patients, and even below 0.5 mSv in most cases. In addition, despite lower effective radiation doses, PC-CT yielded a higher subjective image quality, vascular contrast rating, and quantitative CNR compared with EID-CT.

In adults, several studies demonstrated a higher CNR with a lower radiation dose in thorax CT angiographies, for example, for the diagnosis of pulmonary embolism [21,22]. Another approach in adults yielded a higher CNR with lower doses of contrast agents when scanning the aorta with an effective radiation dose equivalent to EID-CT [23].

In pediatrics, only two studies assessed ECG-gated PC-CT in patients with CHD. Dirrichs et al. [16] demonstrated a higher SNR and CNR in the PC-CT versus a dual-source EID-CT group with an equivalent radiation dose. However, PC-CT examinations were carried out using a tube potential of 90 kVp, compared with 70 kVp in our study. Still, SNR and CNR were higher than in this study (42.3 versus 22.5, and 76.3 versus 39.1, respectively). This might be due to the higher slice thickness and different contrast injection protocol. We prefer a lower-concentrated iodinated contrast agent (300 versus 370 mg/mL) for manual injection due to its lower viscosity. Moreover, we accept a longer post-injection delay to facilitate that the injecting physician may leave the scanner room for radiation protection reasons. Of note, both in the aforementioned study and in our study, inter-observer agreement for the subjective image quality variables was only moderate, whereas it was excellent for SNR and CNR in our study. The moderate agreement for the assessment of the qualitative variables might be at least partially explained by the different levels of experience of the observers in both studies. This underlines the need for objective quantitative measures such as the SNR and CNR.

Stålhammar et al. [17] reported a high subjective image quality at both 70 and 90 kVp, with a lower dose at 70 kVp. This was in line with our data, as 70 kVp PC-CT yielded excellent image quality and contrast at a very low radiation dose. When comparing our results with the aforementioned studies, the age distribution has to be taken into account. In our study, we also included older children and adolescents, while Dirrichs et al. [16] only included neonates and small children (the majority of participants were below 1 year of age). Still, mean effective dose values in our subgroup of patients up to 24 months of age correspond to the aforementioned study (0.53 versus 0.5 mSv). Stålhammar et al. [17] also included adolescents in their study, and reported a mean effective dose of 0.3 mSv using a 70 kVp-protocol and 0.39 mSv using a 90 kVp-protocol. This is slightly lower than in our study, most probably due to the lower mean age and exclusive use of low-kVp protocols, which were also applied in adolescents. In contrast, we used 120 or 140 kV multi-energy spectral acquisition modes in adolescents to account for different body compositions in this group [24], irrespective of the body mass index (which had a wide range between 12.8 and 27.9 kg/m^2^ in subgroup 3). In addition, this mode allows for the reduction of metal artefacts and better stent lumen delineation [25], for example, in patients with aortic coarctation. However, this might have led to higher doses compared with what was published for dual-source scanners [26]. Thus, more research is necessary to compare low- versus high-kVp protocols, especially in the adolescent age group.

Recently, Hellms et al. [27] published a study to compare ultra-low-dose non-ECG-gated PC-CT in small children with a matched cohort of patients examined on a third-generation dual-source EID-CT scanner. The authors report a mean effective dose of 0.11 mSv for their PC-CT cohort (versus 0.51 mSv for subgroup 1 in our study); however, lower conversion factors were used. Still, the DLP was also significantly lower (4.06 mGy*cm versus 16.08 mGy*cm for subgroup 1 in our study). Consequently, despite a bolus tracking technique being used, this led to a lower CNR and SNR, as well as subjective image quality compared with our rather conservative initial dose approach. Further research is needed to assess if this leads to a decrease in diagnostic accuracy or can be tolerated in accordance with the ALARA principle.

When comparing our dose values with studies using a third-generation dual-source CT, the results are inconsistent. Despite a higher age, the mean dose applied in our youngest patients (0–24 months) was lower (0.53 versus 0.66 mSv) than in a collective of 172 scans using dual-source CT [28]. Another study reported lower dose values with a mean of 0.2 mSv in a collective of 27 patients [29]. However, SNR (16.2 versus 22.5) and CNR (18.2 versus 39.1) were noticeably lower compared with our results, most probably due to the 40 keV VMIs used in our study.

### Limitations

Our study has several limitations. First, the sample size is moderate, given the novelty of the PC-CT technology. Second, our EID-CT cohort was scanned with a 256-slice single-source multi-detector EID-CT, which is not the immediate predecessor of the PC-CT system. In this context, the lower possible tube potential of 70 kVp on the PC-CT versus 80 kVp on the EID-CT explains parts of the dose reduction. Compared with conventional dual-source high-pitch EID-CT, as used for example by Dirrichs et al. [16], dose reduction possibilities when performing PC-CT are inherently lower. However, nowadays, many conventional single-source EID-CTs are still in use, even in pediatric cardiovascular radiology. Therefore, these comparisons are of scientific value to demonstrate the advantages of the new PC-CT technology, compared with older scanners. Finally, there is a lack of evidence in the literature on whether to use high-kVp multi-energy spectral acquisition modes in the adolescent group or not, which evidently affects the effective dose. We also did not analyze the inherent spectral information for tissue characterization, iodine mapping, etc.

## 5. Conclusions

In conclusion, our results demonstrate a highly significant dose reduction when using PC-CT for the diagnostic work-up of pediatric patients (in all age groups) with CHD compared with a single-source multi-detector EID-CT. In addition, PC-CT, especially with low-keV VMIs, offered a better subjective image quality, contrast rating, and quantitative CNR. Thus, for radiation protection reasons, PC-CT should be the system of choice for pediatric patients with CHD, if available.

## Figures and Tables

**Table 1:** Technical data on the CT acquisition and image reconstruction parameters.

	EID-CT	PC-CT
Manufacturer	Philips Healthcare	Siemens Healthineers
Model	Brilliance iCT 256	Naeotom Alpha
Software version	iPatient v4.1	Syngo CT VA50A
Single collimation	0.625 mm	0.4 mm
Total collimation	80 mm	57.6 mm
Tube voltage	80 kVp	70 kVp (<10 years) 120/140 kVp (≥10 years)
Pitch factor	Not applicable	3.2
Revolution time	0.27 s	0.25 s
Iterative reconstruction	iDose (4)	QIR-3
Convolution kernel	XCB	Bv40, Bv44
Slice thickness	0.8 mm	0.4 mm (0.8 mm reconstructions used for SNR and CNR assessments)

**Table 2:** Patient characteristics and differences between the EID-CT and PC-CT groups, as well as for the age-based subgroups.

All Patients	EID-CT (n = 16)	PC-CT (n = 27)	*p* Value
age [months] - mean ± SD - minimum/maximum	70 ± 77 0–193	54 ± 60 0–200	0.85
body surface area [m^2^] - mean ± SD - minimum/maximum	0.7 ± 0.52 0.17–1.9	0.69 ± 0.48 0.19–1.84	0.99
body weight [kg] - mean ± SD - minimum/maximum	18.8 ± 18.4 2.6–69	18.9 ± 18.9 2.7–68.8	0.92
body mass index [kg/m^2^] - mean ± SD - minimum/maximum	14.4 ± 2.56 10.8–20.6	15.7 ± 3.57 10.8–27.9	0.2
gender * - male - female	6 10	16 11	0.215
**subgroup 1** **(patients 0–24 months)**	**EID-CT (n = 7)**	**PC-CT (n = 13)**	***p*** **value**
age [months] - mean ± SD - minimum/maximum	3.7 ± 4.5 0–13	7.2 ± 7.1 0–22	0.275
body mass index [kg/m^2^] - mean ± SD - minimum/maximum	12.9 ± 1.61 10.8–15.6	14.5 ± 2.46 10.8–18.9	0.115
gender * - male - female	07	7 6	**0.044**
**subgroup 2** **(patients 2–9 years)**	**EID-CT (n = 4)**	**PC-CT (n = 9)**	***p*** **value**
age [months] - mean ± SD - minimum/maximum	63 ± 20 39–100	59 ± 36 25–109	0.71
body mass index [kg/m^2^] - mean ± SD - minimum/maximum	15.2 ± 1.75 12.6–18.2	14.8 ± 1.21 13.8–16.5	0.503
gender * - male - female	3 1	6 3	1
**subgroup 3** **(patients 10–17 years)**	**EID-CT (n = 5)**	**PC-CT (n = 5)**	***p*** **value**
age [months] - mean ± SD - minimum/maximum	171 ± 23 146–193	159 ± 34 123–200	0.69
body mass index [kg/m^2^] - mean ± SD - minimum/maximum	16.3 ± 3.35 12.8–20.6	19.8 ± 5.69 14.2–27.9	0.31
gender * - male - female	3 2	3 2	1

* 1 female patient had two EID-CT scans, 1 male patient had two PC-CT scans, and 1 female patient had one EID-CT and one PC-CT scan during the study period.

## Data Availability

A.L. and G.S. had full access to all the data in the study and take responsibility for the integrity of the data and the accuracy of the data analysis. The data presented in this study are available on request from the corresponding author. The data are not publicly available due to privacy concerns (medical data).

## Abbreviations

The following abbreviations are used in this manuscript:

CHD	congenital heart disease
CNR	contrast-to-noise ratio
CTDI_vol_	Computed tomography dose index-volume
DLP	dose length product
ECG	electrocardiogram
EID-CT	energy-integrating detector computed tomography
PC-CT	photon-counting computed tomography
ROI	region of interest
SNR	signal-to-noise ratio
VMI	virtual monoenergetic image
